# Supplementary material for: Stigma as a barrier to addressing childhood trauma in conversation with trauma survivors: A study in the general population
Source: PLoS One. 2021 Oct 18;16(10):e0258782. doi: 10.1371/journal.pone.0258782 (PMC8523057; doi:10.1371/journal.pone.0258782)
Supplement: S1 Table — Results of the principal components analysis with varimax rotation and without Kaiser-normalization. h2 communality. (DOCX) [file pone.0258782.s002.docx]

**S1 Table: Rotated factor loadings of stereotype items*.***

| **People who have experienced…** | **Factor negative stereotypes** | **Factor positive stereotypes** | ***h*²** |
| --- | --- | --- | --- |
| … must have already been vulnerable before the event, should they develop a mental illness. | .62 | .09 | .39 |
| …have a higher risk of becoming a criminal. | .60 | -.20 | .40 |
| …are to some extent guilty of what has happened to them. | .64 | -.05 | .42 |
| …are unable to have a stable relationship. | .65 | -.11 | .43 |
| …are unpredictable. | .64 | -.21 | .46 |
| …are able to have good friendships. | -.11 | .68 | .48 |
| …are just as suitable for a responsible job like any other person. | -.08 | .70 | .50 |
| …perform their parental duties just as well as other people. | -.15 | .70 | .51 |
| …have survived a crisis and have grown through it. | .05 | .59 | .35 |
