# Supplementary material for: Stigma as a barrier to addressing childhood trauma in conversation with trauma survivors: A study in the general population
Source: PLoS One. 2021 Oct 18;16(10):e0258782. doi: 10.1371/journal.pone.0258782 (PMC8523057; doi:10.1371/journal.pone.0258782)
Supplement: S2 Table — Global model fit indices of the mediation model in the full sample (single-group structural equation modeling—SGSEM) and with vignette as group factor (multi-group—MGSEM) using a weighted least squares mean- and variance-adjusted estimator and pairwise or listwise deletion of missing values, respectively. A modified variant of the SGSEM, without the indicators “must have been vulnerable already” and “accept as colleague” is also shown. CFI comparative fit index, CI confidence interval, df degrees of freedom, N sample size, RMSEA root mean square error of approximation, SRMR standardized root mean square residual. (DOCX) [file pone.0258782.s003.docx]

**S3 Table: Global model fit of the mediation model.**

| **Model** | **Model specifics** | ***N*** | **χ² (robust)** | ***df*** | **χ²/df** | ***p*(χ²)** | **RMSEA (90% CI)** | ***p*(RMSEA ≤ .05** | **CFI** | **SRMR** |
| --- | --- | --- | --- | --- | --- | --- | --- | --- | --- | --- |
| SGSEM | Pairwise deletion of missings | 1320 | 297.99 | 99 | 3.01 | <.001 | .039  (.034, .044) | 1.00 | .934 | .032 |
|  | Listwise deletion of missings | 1113 | 275.12 | 99 | 2.78 | <.001 | .040  (.034, .046) | 1.00 | .932 | .032 |
|  | Deletion of “predis-posed” & “colleague” | 1320 | 164.11 | 72 | 2.28 | <.001 | .031  (.025, .037) | 1.00 | .961 | .027 |
| MGSEM | Pairwise deletion of missings | 330, 329, 330, 331 | 653.90 | 396 | 1.65 | <.001 | .045  (.038, .051) | .93 | .910 | .049 |
|  | Listwise deletion of missings | 276, 276,  278, 283 | 617.47 | 396 | 1.56 | <.001 | .045  (.038, .052) | .89 | .909 | .049 |
