# Supplementary material for: Stigma as a barrier to addressing childhood trauma in conversation with trauma survivors: A study in the general population
Source: PLoS One. 2021 Oct 18;16(10):e0258782. doi: 10.1371/journal.pone.0258782 (PMC8523057; doi:10.1371/journal.pone.0258782)
Supplement: S3 Table — a. Residual variance-covariance matrix for the SGSEM. Unstandardized residuals of the mediation model in the full sample using single-group structural equation modeling (SGSEM) with a weighted least squares mean- and variance-adjusted estimator and pairwise deletion of missing values. b. Residual variance-covariance matrix for the MGSEM–childhood sexual trauma subgroup. Unstandardized residuals of the childhood sexual trauma vignette group estimated using a multi-group structural equation model (MGSEM) with a weighted least squares mean- and variance-adjusted estimator and pairwise deletion of missing values. c. Residual variance-covariance matrix for the MGSEM–childhood physical trauma subgroup. Unstandardized residuals of the childhood physical trauma vignette group estimated using a multi-group structural equation model (MGSEM) with a weighted least squares mean- and variance-adjusted estimator and pairwise deletion of missing values. d. Residual variance-covariance matrix for the MGSEM–childhood accident trauma subgroup. Unstandardized residuals of the childhood accident trauma vignette group estimated using a multi-group structural equation model (MGSEM) with a weighted least squares mean- and variance-adjusted estimator and pairwise deletion of missing values. e. Residual variance-covariance matrix for the MGSEM–adult physical trauma subgroup. Unstandardized residuals of the adult physical trauma vignette group estimated using a multi-group structural equation model (MGSEM) with a weighted least squares mean- and variance-adjusted estimator and pairwise deletion of missing values. (DOCX) [file pone.0258782.s004.docx]

**S4 Table a: Residual variance-covariance matrix for the SGSEM.**

|  | Predis-posed | Unpre-dictable | Crimi-nal risk | Guilty | Rela-tion-ship | Sub-tenant | Col-league | Child care | Mar-riage | Friends | Recom-menda-tion | Friend-ships | Job | Parent-hood | Perso-nal growth | Taboo |
| --- | --- | --- | --- | --- | --- | --- | --- | --- | --- | --- | --- | --- | --- | --- | --- | --- |
| Predisposed | 1.76 |  |  |  |  |  |  |  |  |  |  |  |  |  |  |  |
| Unpre-dictable | 0.40 | 1.50 |  |  |  |  |  |  |  |  |  |  |  |  |  |  |
| Criminal risk | 0.28 | 0.57 | 1.64 |  |  |  |  |  |  |  |  |  |  |  |  |  |
| Guilty | 0.35 | 0.37 | 0.35 | 1.25 |  |  |  |  |  |  |  |  |  |  |  |  |
| Relationship | 0.43 | 0.41 | 0.45 | 0.42 | 1.63 |  |  |  |  |  |  |  |  |  |  |  |
| Subtenant | 0.22 | 0.19 | 0.14 | 0.20 | 0.25 | 1.85 |  |  |  |  |  |  |  |  |  |  |
| Colleague | 0.20 | 0.16 | 0.13 | 0.21 | 0.17 | 0.46 | 0.74 |  |  |  |  |  |  |  |  |  |
| Child care | 0.23 | 0.27 | 0.20 | 0.19 | 0.17 | 0.85 | 0.35 | 1.79 |  |  |  |  |  |  |  |  |
| Marriage | 0.27 | 0.26 | 0.23 | 0.26 | 0.23 | 0.69 | 0.44 | 0.79 | 1.41 |  |  |  |  |  |  |  |
| Friends | 0.28 | 0.21 | 0.18 | 0.28 | 0.19 | 0.69 | 0.45 | 0.66 | 0.80 | 1.18 |  |  |  |  |  |  |
| Recommen-dation | 0.12 | 0.19 | 0.15 | 0.17 | 0.10 | 0.61 | 0.34 | 0.67 | 0.63 | 0.70 | 1.30 |  |  |  |  |  |
| Friendships | -0.06 | -0.23 | -0.18 | -0.13 | -0.18 | -0.33 | -0.21 | -0.33 | -0.37 | -0.33 | -0.27 | 1.07 |  |  |  |  |
| Job | -0.05 | -0.22 | -0.14 | -0.13 | -0.16 | -0.27 | -0.28 | -0.34 | -0.38 | -0.30 | -0.34 | 0.39 | 1.05 |  |  |  |
| Parenthood | -0.12 | -0.22 | -0.25 | -0.13 | -0.21 | -0.32 | -0.22 | -0.39 | -0.38 | -0.30 | -0.26 | 0.33 | 0.36 | 1.06 |  |  |
| Personal growth | 0.04 | -0.10 | -0.15 | -0.03 | -0.07 | -0.18 | -0.12 | -0.20 | -0.20 | -0.19 | -0.16 | 0.20 | 0.18 | 0.28 | 0.95 |  |
| Taboo | 0.04 | 0.04 | 0.02 | 0.00 | 0.03 | -0.24 | -0.10 | -0.20 | -0.14 | -0.17 | -0.16 | 0.09 | 0.05 | 0.03 | 0.03 | 1.00 |

**S4 Table b: Residual variance-covariance matrix for the MGSEM – childhood sexual trauma subgroup.**

|  | Predis-posed | Unpre-dictable | Crimi-nal risk | Guilty | Rela-tion-ship | Sub-tenant | Col-league | Child care | Mar-riage | Friends | Recom-menda-tion | Friend-ships | Job | Parent-hood | Perso-nal growth | Taboo |
| --- | --- | --- | --- | --- | --- | --- | --- | --- | --- | --- | --- | --- | --- | --- | --- | --- |
| Predisposed | 0.00 |  |  |  |  |  |  |  |  |  |  |  |  |  |  |  |
| Unpre-dictable | -0.11 | 0.00 |  |  |  |  |  |  |  |  |  |  |  |  |  |  |
| Criminal risk | 0.11 | 0.02 | 0.00 |  |  |  |  |  |  |  |  |  |  |  |  |  |
| Guilty | -0.02 | -0.04 | 0.00 | 0.00 |  |  |  |  |  |  |  |  |  |  |  |  |
| Relationship | 0.25 | -0.05 | 0.03 | 0.05 | 0.00 |  |  |  |  |  |  |  |  |  |  |  |
| Subtenant | -0.11 | 0.07 | -0.09 | 0.07 | 0.06 | 0.00 |  |  |  |  |  |  |  |  |  |  |
| Colleague | 0.06 | -0.03 | 0.03 | 0.10 | 0.07 | 0.03 | 0.00 |  |  |  |  |  |  |  |  |  |
| Child care | 0.09 | 0.19 | 0.01 | -0.05 | -0.09 | 0.09 | -0.15 | 0.00 |  |  |  |  |  |  |  |  |
| Marriage | 0.01 | 0.09 | 0.01 | -0.03 | -0.01 | -0.03 | 0.01 | 0.05 | 0.00 |  |  |  |  |  |  |  |
| Friends | 0.07 | -0.02 | -0.08 | 0.01 | -0.06 | 0.02 | 0.00 | -0.03 | -0.04 | 0.00 |  |  |  |  |  |  |
| Recommen-dation | -0.07 | -0.03 | -0.08 | -0.04 | -0.19 | 0.14 | -0.02 | 0.07 | 0.00 | 0.09 | 0.00 |  |  |  |  |  |
| Friendships | 0.05 | -0.09 | 0.04 | -0.02 | -0.05 | 0.01 | 0.04 | -0.01 | 0.03 | -0.01 | 0.06 | 0.00 |  |  |  |  |
| Job | 0.12 | -0.01 | 0.00 | 0.01 | 0.07 | 0.13 | -0.10 | 0.00 | -0.03 | -0.03 | -0.04 | 0.00 | 0.00 |  |  |  |
| Parenthood | -0.04 | 0.00 | -0.15 | 0.02 | -0.06 | 0.09 | -0.01 | -0.05 | -0.04 | 0.00 | 0.07 | 0.04 | -0.07 | 0.00 |  |  |
| Personal growth | 0.11 | 0.07 | -0.09 | 0.10 | 0.02 | 0.07 | 0.02 | -0.04 | 0.04 | -0.01 | -0.01 | 0.01 | 0.00 | 0.09 | 0.00 |  |
| Taboo | 0.00 | 0.01 | -0.12 | 0.00 | 0.10 | -0.07 | 0.06 | -0.10 | 0.06 | 0.05 | -0.08 | 0.01 | 0.00 | 0.01 | -0.05 | 0.00 |

**S4 Table c: Residual variance-covariance matrix for the MGSEM – childhood physical trauma subgroup.**

|  | Predis-posed | Unpre-dictable | Crimi-nal risk | Guilty | Rela-tion-ship | Sub-tenant | Col-league | Child care | Mar-riage | Friends | Recom-menda-tion | Friend-ships | Job | Parent-hood | Perso-nal growth | Taboo |
| --- | --- | --- | --- | --- | --- | --- | --- | --- | --- | --- | --- | --- | --- | --- | --- | --- |
| Predisposed | 0.00 |  |  |  |  |  |  |  |  |  |  |  |  |  |  |  |
| Unpre-dictable | 0.10 | 0.00 |  |  |  |  |  |  |  |  |  |  |  |  |  |  |
| Criminal risk | -0.14 | 0.18 | 0.00 |  |  |  |  |  |  |  |  |  |  |  |  |  |
| Guilty | 0.01 | 0.01 | -0.08 | 0.00 |  |  |  |  |  |  |  |  |  |  |  |  |
| Relationship | -0.08 | -0.02 | 0.06 | 0.03 | 0.00 |  |  |  |  |  |  |  |  |  |  |  |
| Subtenant | 0.10 | -0.10 | -0.12 | -0.06 | -0.04 | 0.00 |  |  |  |  |  |  |  |  |  |  |
| Colleague | 0.07 | -0.05 | -0.06 | 0.04 | -0.03 | 0.05 | 0.00 |  |  |  |  |  |  |  |  |  |
| Child care | 0.09 | -0.05 | -0.03 | -0.10 | -0.07 | 0.28 | -0.02 | 0.00 |  |  |  |  |  |  |  |  |
| Marriage | 0.15 | -0.06 | -0.07 | 0.08 | -0.03 | -0.11 | -0.02 | 0.03 | 0.00 |  |  |  |  |  |  |  |
| Friends | 0.15 | -0.05 | 0.01 | 0.13 | 0.01 | -0.03 | -0.04 | -0.07 | 0.13 | 0.00 |  |  |  |  |  |  |
| Recommen-dation | 0.05 | 0.07 | 0.07 | 0.01 | -0.03 | -0.05 | -0.07 | 0.06 | 0.02 | 0.19 | 0.00 |  |  |  |  |  |
| Friendships | 0.10 | 0.04 | 0.03 | 0.03 | -0.08 | 0.03 | -0.06 | 0.02 | -0.04 | 0.00 | 0.01 | 0.00 |  |  |  |  |
| Job | 0.08 | -0.05 | 0.04 | 0.06 | -0.03 | 0.00 | -0.12 | -0.04 | 0.03 | 0.07 | -0.02 | 0.02 | 0.00 |  |  |  |
| Parenthood | 0.06 | -0.01 | -0.19 | -0.01 | -0.06 | -0.03 | 0.00 | 0.01 | -0.02 | 0.00 | 0.07 | -0.08 | -0.03 | 0.00 |  |  |
| Personal growth | 0.16 | 0.02 | -0.08 | 0.05 | -0.06 | 0.02 | -0.02 | 0.02 | 0.03 | 0.07 | 0.06 | 0.03 | -0.01 | 0.12 | 0.00 |  |
| Taboo | 0.04 | -0.08 | 0.11 | -0.02 | -0.02 | -0.19 | 0.04 | 0.05 | 0.04 | 0.03 | -0.02 | 0.05 | -0.02 | -0.02 | 0.00 | 0.00 |

**S4 Table d: Residual variance-covariance matrix for the MGSEM – childhood accident trauma subgroup.**

|  | Predis-posed | Unpre-dictable | Crimi-nal risk | Guilty | Rela-tion-ship | Sub-tenant | Col-league | Child care | Mar-riage | Friends | Recom-menda-tion | Friend-ships | Job | Parent-hood | Perso-nal growth | Taboo |
| --- | --- | --- | --- | --- | --- | --- | --- | --- | --- | --- | --- | --- | --- | --- | --- | --- |
| Predisposed | 0.00 |  |  |  |  |  |  |  |  |  |  |  |  |  |  |  |
| Unpre-dictable | -0.03 | 0.00 |  |  |  |  |  |  |  |  |  |  |  |  |  |  |
| Criminal risk | -0.09 | 0.16 | 0.00 |  |  |  |  |  |  |  |  |  |  |  |  |  |
| Guilty | 0.08 | -0.11 | 0.05 | 0.00 |  |  |  |  |  |  |  |  |  |  |  |  |
| Relationship | 0.04 | -0.07 | 0.03 | 0.08 | 0.00 |  |  |  |  |  |  |  |  |  |  |  |
| Subtenant | 0.15 | -0.10 | -0.10 | -0.05 | 0.08 | 0.00 |  |  |  |  |  |  |  |  |  |  |
| Colleague | 0.14 | 0.07 | 0.03 | 0.07 | 0.12 | 0.05 | 0.00 |  |  |  |  |  |  |  |  |  |
| Child care | 0.05 | -0.03 | -0.04 | 0.05 | -0.01 | 0.03 | -0.08 | 0.00 |  |  |  |  |  |  |  |  |
| Marriage | 0.13 | -0.05 | 0.00 | -0.04 | 0.01 | 0.00 | -0.07 | 0.03 | 0.00 |  |  |  |  |  |  |  |
| Friends | 0.11 | -0.08 | -0.10 | 0.05 | -0.05 | 0.01 | 0.01 | -0.02 | 0.09 | 0.00 |  |  |  |  |  |  |
| Recommen-dation | -0.04 | -0.09 | -0.06 | -0.01 | -0.07 | -0.01 | -0.03 | 0.00 | -0.02 | 0.11 | 0.00 |  |  |  |  |  |
| Friendships | 0.16 | -0.14 | -0.06 | -0.04 | 0.07 | -0.06 | -0.01 | 0.00 | 0.01 | 0.01 | -0.03 | 0.00 |  |  |  |  |
| Job | 0.11 | -0.03 | 0.03 | 0.06 | 0.06 | 0.05 | -0.02 | -0.01 | -0.05 | 0.03 | -0.01 | 0.04 | 0.00 |  |  |  |
| Parenthood | -0.03 | -0.12 | -0.07 | 0.08 | -0.10 | -0.01 | -0.01 | -0.08 | 0.02 | 0.11 | -0.03 | -0.05 | -0.01 | 0.00 |  |  |
| Personal growth | 0.12 | -0.10 | 0.03 | -0.01 | 0.04 | 0.10 | 0.00 | 0.00 | -0.04 | -0.01 | 0.01 | -0.03 | 0.01 | 0.07 | 0.00 |  |
| Taboo | 0.01 | 0.02 | 0.10 | -0.09 | -0.02 | -0.01 | -0.04 | -0.01 | 0.01 | 0.02 | 0.02 | 0.04 | 0.05 | -0.06 | -0.03 | 0.00 |

**S4 Table e: Residual variance-covariance matrix for the MGSEM – adult physical trauma subgroup.**

|  | Predis-posed | Unpre-dictable | Crimi-nal risk | Guilty | Rela-tion-ship | Sub-tenant | Col-league | Child care | Mar-riage | Friends | Recom-menda-tion | Friend-ships | Job | Parent-hood | Perso-nal growth | Taboo |
| --- | --- | --- | --- | --- | --- | --- | --- | --- | --- | --- | --- | --- | --- | --- | --- | --- |
| Predisposed | 0.00 |  |  |  |  |  |  |  |  |  |  |  |  |  |  |  |
| Unpre-dictable | 0.15 | 0.00 |  |  |  |  |  |  |  |  |  |  |  |  |  |  |
| Criminal risk | -0.12 | 0.09 | 0.00 |  |  |  |  |  |  |  |  |  |  |  |  |  |
| Guilty | 0.02 | -0.05 | -0.06 | 0.00 |  |  |  |  |  |  |  |  |  |  |  |  |
| Relationship | 0.13 | -0.01 | -0.03 | -0.03 | 0.00 |  |  |  |  |  |  |  |  |  |  |  |
| Subtenant | 0.00 | -0.08 | 0.05 | -0.04 | 0.05 | 0.00 |  |  |  |  |  |  |  |  |  |  |
| Colleague | 0.06 | 0.02 | -0.03 | 0.11 | 0.00 | -0.04 | 0.00 |  |  |  |  |  |  |  |  |  |
| Child care | -0.07 | -0.06 | 0.01 | -0.03 | -0.05 | 0.26 | -0.11 | 0.00 |  |  |  |  |  |  |  |  |
| Marriage | 0.00 | -0.01 | 0.06 | 0.12 | 0.02 | -0.05 | -0.03 | 0.03 | 0.00 |  |  |  |  |  |  |  |
| Friends | 0.05 | -0.05 | 0.00 | 0.10 | 0.00 | 0.00 | 0.06 | -0.05 | 0.01 | 0.00 |  |  |  |  |  |  |
| Recommen-dation | -0.10 | -0.04 | -0.05 | -0.05 | -0.01 | -0.03 | -0.02 | 0.14 | -0.03 | 0.01 | 0.00 |  |  |  |  |  |
| Friendships | 0.00 | -0.01 | -0.06 | 0.10 | 0.00 | -0.01 | 0.01 | 0.01 | -0.04 | -0.01 | -0.01 | 0.00 |  |  |  |  |
| Job | 0.03 | -0.08 | -0.04 | 0.00 | -0.12 | 0.12 | 0.00 | 0.08 | 0.00 | 0.09 | -0.10 | 0.08 | 0.00 |  |  |  |
| Parenthood | 0.05 | -0.06 | -0.01 | 0.00 | 0.04 | 0.01 | 0.00 | -0.06 | -0.05 | 0.07 | 0.04 | 0.00 | 0.08 | 0.00 |  |  |
| Personal growth | 0.06 | 0.00 | -0.09 | 0.07 | 0.15 | -0.18 | -0.01 | -0.01 | -0.02 | -0.04 | -0.06 | -0.08 | -0.06 | 0.00 | 0.00 |  |
| Taboo | 0.01 | 0.10 | 0.04 | -0.11 | -0.04 | -0.03 | -0.01 | -0.04 | 0.10 | -0.02 | -0.01 | -0.02 | 0.02 | -0.02 | 0.03 | 0.00 |
