# Supplementary material for: Stigma as a barrier to addressing childhood trauma in conversation with trauma survivors: A study in the general population
Source: PLoS One. 2021 Oct 18;16(10):e0258782. doi: 10.1371/journal.pone.0258782 (PMC8523057; doi:10.1371/journal.pone.0258782)
Supplement: S4 Table — Global fit indices for the measurement model (i.e. latent variables and their indicator variables) and their change (Δ) when the factor loadings (metric invariance model) and intercepts (scalar invariance model) of the indicator variables are constrained to be equal across all four groups. For comparability with the full multi-group structural equation model a weighted least squares estimator and pairwise deletion of missing values was used. CFI comparative fit index, RMSEA root mean square error of approximation, SRMR standardized root mean square residual. *** p < .001. (DOCX) [file pone.0258782.s005.docx]

**S5 Table: Measurement invariance tests of the measurement model.**

| **Type of invariance** | **χ² (Δ)** | ***df* (Δ)** | ***p*(Δχ²)** | **CFI (Δ)** | **RMSEA (Δ)** | **SRMR (Δ)** |
| --- | --- | --- | --- | --- | --- | --- |
| Configural | 335.84 | 348 |  | .957 | .036 | .046 |
| Metric | 421.02 (38.83) | 384 (36) | .34 | .964 (.007) | .031 (-.005) | .052 (.006) |
| Scalar | 507.86 (87.94) | 420 (36) | < .001*** | .948 (-.017) | .036 (.005) | .056 (.004) |
