# Supplementary material for: Stigma as a barrier to addressing childhood trauma in conversation with trauma survivors: A study in the general population
Source: PLoS One. 2021 Oct 18;16(10):e0258782. doi: 10.1371/journal.pone.0258782 (PMC8523057; doi:10.1371/journal.pone.0258782)
Supplement: Attachment — Submitted filename: PONE-D-21-09346_reviewer.pdf [file pone.0258782.s006.pdf]

PLOS ONE

Stigma as a barrier to addressing childhood trauma in conversation with trauma survivors: a study in the general population

--Manuscript Draft--

**Manuscript Number:**	PONE-D-21-09346
**Article Type:**	Research Article
**Full Title:**	Stigma as a barrier to addressing childhood trauma in conversation with trauma survivors: a study in the general population
**Short Title:**	Stigma and childhood trauna
**Corresponding Author:**	Georg Schomerus<br>University Hospital Leipzig: Universitatsklinikum Leipzig<br>Leipzig, GERMANY
**Keywords:**	stigma; childhood trauma; representative population survey
**Abstract:**	Victims of childhood trauma report shame and anticipation of stigma, leading to non-disclosure and avoidance of help. Stigma is potentially aggravating the mental health consequences of childhood trauma. So far the stigma of childhood trauma has not been examined, and it is unclear whether it interferes with reaching out to affected individuals. In a representative sample of the German general population (N=1320; 47.7% male) respondents' willingness to reach out to trauma victims was assessed with brief case vignettes pertaining to past childhood sexual/ physical abuse or accidents, and adult physical abuse. We further elicited social distance, stereotypes, contact and own traumatic experiences. We found that the willingness to reach out differed by the type of trauma. Of the respondents, 45% indicated they were unlikely to reach out to a victim of past childhood sexual abuse, 38% to a victim of child physical abuse, 31% to someone reporting a childhood accident and 25% to someone reporting adult physical abuse. Negative stereotypes showed different patterns for each scenario, with childhood sexual and physical abuse eliciting particularly strong negative reactions. Negative stereotypes were related to more desire for social distance. Social distance, in turn, was related to lower likelihood to reach out to someone having previously experienced childhood sexual and physical abuse; structural equation modeling confirmed social distance as mediator of the relationship between negative stereotypes and willingness to reach out in sensitivity analysis. Our analyses further revealed an ambiguous role of negative stereotypes in approaching trauma victims, which has yet to be examined in future studies. Conclusions: There is evidence for a stigma associated with having survived childhood trauma, which is interfering with offering help. Other barriers are likely also contributing to the taboo surrounding adverse childhood experiences and warrant further investigation.
**Order of Authors:**	Georg Schomerus
	Theresia Rechenberg
	Tobias Gfesser
	Hans J Grabe
	Mario Liebergesell
	Christian Sander
	Christine Ulke
	Stephanie Schindler
	Sven Speerforck
**Additional Information:**	
**Question**	**Response**
**Financial Disclosure**	The authors received no specific funding for this work

Powered by Editorial Manager® and ProduXion Manager® from Aries Systems Corporation

Enter a financial disclosure statement that describes the sources of funding for the work included in this submission. Review the submission guidelines for detailed requirements. View published research articles from *PLOS ONE* for specific examples.<br><br>This statement is required for submission and **will appear in the published article** if the submission is accepted. Please make sure it is accurate.<br><br>**Unfunded studies**<br>Enter: *The author(s) received no specific funding for this work.*<br><br>**Funded studies**<br>Enter a statement with the following details:<br>• Initials of the authors who received each award<br>• Grant numbers awarded to each author<br>• The full name of each funder<br>• URL of each funder website<br>• Did the sponsors or funders play any role in the study design, data collection and analysis, decision to publish, or preparation of the manuscript?<br>• **NO** - Include this sentence at the end of your statement: *The funders had no role in study design, data collection and analysis, decision to publish, or preparation of the manuscript.*<br>• **YES** - Specify the role(s) played.<br><br>* typeset	
**Competing Interests**<br><br>Use the instructions below to enter a competing interest statement for this submission. On behalf of all authors, disclose any competing interests that could be perceived to bias this work—acknowledging all financial support and any other relevant financial or non-financial competing interests.<br><br>This statement is required for submission and **will appear in the published article** if the submission is accepted. Please make	The authors have declared that no competing interests exist.

sure it is accurate and that any funding sources listed in your Funding Information later in the submission form are also declared in your Financial Disclosure statement.<br><br>View published research articles from *PLOS ONE* for specific examples.<br><br>**NO authors have competing interests**<br><br>Enter: *The authors have declared that no competing interests exist.*<br><br>**Authors with competing interests**<br><br>Enter competing interest details beginning with this statement:<br><br>*I have read the journal's policy and the authors of this manuscript have the following competing interests: [insert competing interests here]*<br><br>* typeset	
**Ethics Statement**<br><br>Enter an ethics statement for this submission. This statement is required if the study involved:<br><br>• Human participants<br>• Human specimens or tissue<br>• Vertebrate animals or cephalopods<br>• Vertebrate embryos or tissues<br>• Field research<br><br>Write "N/A" if the submission does not require an ethics statement.<br><br>General guidance is provided below. Consult the submission guidelines for detailed instructions. **Make sure that all information entered here is included in the Methods section of the manuscript.**	The University of Leipzig Medical Center Review Board approved of this study. Informed consent for the CATI was obtained orally.

**Format for specific study types**<br><br>**Human Subject Research (involving human participants and/or tissue)**<br>• Give the name of the institutional review board or ethics committee that approved the study<br>• Include the approval number and/or a statement indicating approval of this research<br>• Indicate the form of consent obtained (written/oral) or the reason that consent was not obtained (e.g. the data were analyzed anonymously)<br><br>**Animal Research (involving vertebrate animals, embryos or tissues)**<br>• Provide the name of the Institutional Animal Care and Use Committee (IACUC) or other relevant ethics board that reviewed the study protocol, and indicate whether they approved this research or granted a formal waiver of ethical approval<br>• Include an approval number if one was obtained<br>• If the study involved *non-human primates*, add *additional details* about animal welfare and steps taken to ameliorate suffering<br>• If anesthesia, euthanasia, or any kind of animal sacrifice is part of the study, include briefly which substances and/or methods were applied<br><br>**Field Research**<br><br>Include the following details if this study involves the collection of plant, animal, or other materials from a natural setting:<br>• Field permit number<br>• Name of the institution or relevant body that granted permission	
**Data Availability**<br><br>Authors are required to make all data underlying the findings described fully available, without restriction, and from the time of publication. PLOS allows rare exceptions to address legal and ethical concerns. See the PLOS Data Policy and FAQ for detailed information.	Yes - all data are fully available without restriction

A Data Availability Statement describing where the data can be found is required at submission. Your answers to this question constitute the Data Availability Statement and **will be published in the article**, if accepted.<br><br>**Important:** Stating 'data available on request from the author' is not sufficient. If your data are only available upon request, select 'No' for the first question and explain your exceptional situation in the text box.<br><br>Do the authors confirm that all data underlying the findings described in their manuscript are fully available without restriction?	
**Describe where the data may be found in full sentences. If you are copying our sample text, replace any instances of XXX with the appropriate details.**<br><br>• If the data are **held or will be held in a public repository**, include URLs, accession numbers or DOIs. If this information will only be available after acceptance, indicate this by ticking the box below. For example: *All XXX files are available from the XXX database (accession number(s) XXX, XXX.).*<br>• If the data are all contained **within the manuscript and/or Supporting Information files**, enter the following: *All relevant data are within the manuscript and its Supporting Information files.*<br>• If neither of these applies but you are able to provide **details of access elsewhere**, with or without limitations, please do so. For example:<br><br>*Data cannot be shared publicly because of [XXX]. Data are available from the XXX Institutional Data Access / Ethics Committee (contact via XXX) for researchers who meet the criteria for access to confidential data.*<br><br>*The data underlying the results presented in the study are available from (include the name of the third party*	All data files are available from the XXX database (accession number(s) XXX, XXX.)

and contact information or URL).<br>• This text is appropriate if the data are owned by a third party and authors do not have permission to share the data.<br><br>* typeset	
Additional data availability information:	Tick here if the URLs/accession numbers/DOIs will be available only after acceptance of the manuscript for publication so that we can ensure their inclusion before publication.

Powered by Editorial Manager® and ProduXion Manager® from Aries Systems Corporation

Manuscript Click here to access/download;Manuscript;Stigma of child abuse plosone final.docx

Stigma as a barrier to addressing childhood trauma in conversation with trauma survivors: a study in the general population

Georg Schomerus[1]*, Theresia Rechenberg[1], Tobias Gfesser[1], Hans Grabe[2], Mario Liebergesell[1], Christian Sander[1], Stephanie Schindler[1], Christine Ulke[1], Sven Speerforck[1]

[1] Department of Psychiatry and Psychotherapy, University of Leipzig Medical Center, Leipzig, Germany
[2] Department of Psychiatry and Psychotherapy, University of Greifswald, Greifswald, Germany

* Corresponding author

E-mail: Georg.schomerus@medizin.uni-leipzig.de (GS)

Abstract

Victims of childhood trauma report shame and anticipation of stigma, leading to non-disclosure and avoidance of help. Stigma is potentially aggravating the mental health consequences of childhood trauma. So far the stigma of childhood trauma has not been examined, and it is unclear whether it interferes with reaching out to affected individuals. In a representative sample of the German general population (N=1320; 47.7% male) respondents' willingness to reach out to trauma victims was assessed with brief case vignettes pertaining to past childhood sexual/ physical abuse or accidents, and adult physical abuse. We further elicited social distance, stereotypes, contact and own traumatic experiences. We found that the willingness to reach out differed by the type of trauma. Of the respondents, 45% indicated they were unlikely to reach out to a victim of past childhood sexual abuse, 38% to a victim of child physical abuse, 31% to someone reporting a childhood accident and 25% to someone reporting adult physical abuse. Negative stereotypes showed different patterns for each scenario, with childhood sexual and physical abuse eliciting particularly strong negative reactions. Negative stereotypes were related to more desire for social distance. Social distance, in turn, was related to lower likelihood to reach out to someone having previously experienced childhood sexual and physical abuse; structural equation modeling confirmed social distance as mediator of the relationship between negative stereotypes and willingness to reach out in sensitivity analysis. Our analyses further revealed an ambiguous role of negative stereotypes in approaching trauma victims, which has yet to be examined in future studies. Conclusions: There is evidence for a stigma associated with having survived childhood trauma, which is interfering with offering help. Other barriers are likely also contributing to the taboo surrounding adverse childhood experiences and warrant further investigation.

Introduction

Childhood trauma (CT) is a well-established causal risk factor for lifetime occurrence of mental disorders (1)(2). Having suffered childhood maltreatment (i.e. physical, sexual or emotional abuse, or emotional or physical neglect) increases the risk of developing not only post-traumatic stress disorder, but several severe mental disorders such as depression (3, 4), borderline-personality disorder (5), substance use disorders, or schizophrenia (6). CT has been described as a "hidden wound" carried on in later life, affecting the lives of victims for decades (1).

There is evidence that experiencing stigma is aggravating the consequences of CT. Victims report shame, self-blame and anticipated stigma, leading to non-disclosure and avoidance of help (7). Experienced and anticipated negative social reactions are associated with poorer outcomes like PTSD, depression and maladaptive coping (7). In children, this has best been studied for child sexual abuse, and less for child physical or emotional abuse and neglect. In adults, negative consequences of stigma experiences have also been reported for women suffering from intimate partner violence (8)(9). A study on long term effects of childhood sexual abuse on sexual difficulties found abuse-specific stigmatization, more than abuse severity, to be associated with later problems (10). So far, emphasis has so far been put on survivors of sexual violence during childhood (7), or of intimate partner violence (8). Stigma has been described as an "attribute that is deeply discrediting" (11) p 13). By increasing social isolation, inducing shame, preventing help-seeking and overall spoiling the social identity of those having experienced CT (11), stigma could well contribute to the adverse effects of CT on mental health.

Stigma is a social process. According to a widely used conceptualization, stigma consists of labeling, stereotyping, separation and status loss (12). The prevalence of stigma on a

population level has been shown to predict the degree of stigma experiences and self-stigma on an individual level (13). Hence, although shame, self-stigma and anticipation of negative reactions occur within a stigmatized person, the root cause of stigma experiences is population attitudes (14). The public stigma of CT could thus pose an area where some of the negative consequences of CT could be understood, addressed and prevented. Improving attitudes towards CT survivors could improve psychosocial outcomes of CT. However, to our knowledge, there are no empirical studies examining public attitudes towards survivors of CT. There are, for example, no studies exploring whether the general population does hold negative views of persons who have experienced CT, and whether they are indeed reluctant to engage with someone with CT, should this person reach out for help.

In this study, we examine the prevalence of negative attitudes towards persons with CT, and whether stigma interferes with reaching out to a person with CT, adding to the overall taboo that surrounds survivors of childhood trauma. Based on a short vignette of a person recounting his or her trauma experience, we elicit the willingness to talk to this person about their experience, aiming to define the taboo surrounding any particular trauma. We further elicit the prevalence of potential negative and positive stereotypes, and the desire for social distance from this person in order to see whether and to what extent stigma and discrimination towards persons with CT exist and whether they interfere with reaching out.

While our first aim is to establish the prevalence of stigma towards persons with CT, we further assume that attitudes differ according to the type of trauma. Based on the literature on individual stigma experiences and from studies on trauma-related mental health problems, we hypothesize that sexual abuse elicits more stigma than physical abuse (H1, (15). Further, we hypothesize that interpersonal trauma is associated with more stigma than accidental trauma (H2, (16)). Since adults are perceived as more stable in order to overcome

traumatic events, while children are regarded as more vulnerable and more profoundly affected by trauma (17), we assume that CT triggers more stigma than adult trauma (H3) . Finally, disentangling stereotypes, stigma and reluctance to reach out to someone with adverse childhood experiences, we expect the following relationships: Negative stereotypes are related to stronger social distance, and both negative stereotypes and social distance are related to stronger reluctance to reach out (H4).

Materials and Methods

Sample

We conducted a population-based telephone survey (computer-assisted telephone interview, CATI), among persons aged 18 and older residing in Germany. Using a "dual-frame approach”, the initial sample was randomly drawn from a combination of registered private telephone numbers and generated numbers, allowing for ex-directory households as well, and an additional share of mobile telephone numbers of 30%. Target persons within households were selected randomly using the Kish-Selection-Grid. Persons who were reached via mobile telephone numbers automatically became target persons. Informed consent was considered to have been given when individuals agreed to complete the interview. The data collected in both modes are combined by a design weighting in which the probabilities of selection were mathematically corrected. Fieldwork was carried out by USUMA (Berlin), a company specialized in market and social research. The study was approved by the Ethics Committee of the Medical Faculty, University of Leipzig. In total, 1320 persons completed the interview, reflecting a response rate of 26.4 %. The sample

contained slightly more women and better educated persons than the general population (see Table 1).

Table 1. Socio-demographic characteristics of the study sample

	Total population of Germany [%]	Sample N=1320 [%]
Gender		
Male	48.8	47.7
Female	51.2	52.0
Diverse	n/a	0.3
Age		
18-29	17.0	10.8
30-39	14.2	11.7
40-49	19.9	13.9
50-64	24.3	31.7
65-74	13.5	15.8
75+	11.2	16.1
Educational attainment		
8-9 y	35.1	15.3
10 y	23.6	28.6
>10 y	41.1	56.1

Population data from the Federal Office of Statistics (Dec. 2019)

Interview and vignettes

At the beginning of the fully structured telephone interview, respondents were presented with a short vignette describing an encounter with a new neighbor, the gender of the person varying at random: *Imagine you have a new neighbor. When talking to you, they indicate that they have experienced [traumatic event] and are still dealing with the consequences. How likely would it be that you actively raise this topic again with your neighbor?* Respondents were randomly assigned to one of 4 versions of the scenario, differing solely by the [traumatic event] mentioned: sexual abuse as a child (n=330), physical abuse as a child (n=329), serious accident as child (n=330), physical abuse as an adult (n=331). Answers were given on a 5-point Likert-scale ranging from 1 “very likely” to 5 “very unlikely”. We inverted the scale and collapsed these answers into three categories by combining the two values of

either side of the midpoint, resulting in a 3-point scale with 3 “willing to talk about the neighbor’s trauma experience”, 2 “undecided”, and 1 “unwilling to talk about the neighbor’s trauma experience”. We conceived this question as an indicator of the taboo surrounding past traumatic experiences.

Social distance

We used a 6-item social distance scale adopted from of a scale developed by (18) that has been used in various vignette based population studies in Germany (e.g., (19). The scale asks whether respondents are willing to interact with the person described in the vignette in various hypothetical everyday situations: rent a room, work together, take care of a young child, have married into family, introduce to friends, recommend for a job (18). Items were rated on a 5-point Likert scale with 1=“very likely” to 5=“very unlikely”. We calculated a mean score for all respondents who had answered at least 4 of 6 items. Higher scores indicate a stronger desire for social distance. Social distance is frequently used as an indicator of individual discrimination.

Stereotypes

We elicited 10 potential stereotypes about a person having experienced the type of trauma mentioned in the vignette. These stereotypes were developed from discussions with people with lived experience and psychotherapists. Statements started with “Persons who have experienced [traumatic event]…”, followed by four positively framed statements (are able to have good friendships; are just as suitable for a responsible job like any other person; perform their parental duties just as well as other people; have survived a crisis and have

grown through it), and six negative statements (are unpredictable; have a higher risk of becoming a criminal; are harmed for the rest of their lives; are guilty of what has happened to them to a certain degree; are not able to have a stable relationship; have already been vulnerable before the event, should they develop a mental illness). Answers had to be given on a 5-point Likert scale, ranging from "1" indicating strong agreement to "5" indicating strong disagreement. Again, we inverted the scale and regrouped the responses into 1 “disagree”, 2 “undecided”, and 3 “agree”.

Contact to people with CT and respondents’ personal experience of childhood adversities

We determined respondents’ previous contacts to persons with traumatic experiences by asking whether they know a person who was affected by a traumatic event as described in the vignette (1=yes). We used two items from the Childhood Trauma Screener (CTS; (20) to assess potential traumatic experiences in the respondents' childhood. Sexual abuse was inquired with “When I was growing up, someone molested me”; physical abuse with “When I was growing up, I got hit so hard by someone in my family that I had to see a doctor or go to the hospital”. (CTQ; (21)). All items are rated on a 5-point Likert scale with 1=“never”, 2= “rarely”, 3=“sometimes”, 4=“often”, 5=“very often”. Following Grabe (20), we coded sexual abuse (0=never) and (1=rarely, sometimes, often and very often); and physical abuse (0= never, rarely) and (1=sometimes, often, very often).

Additionally, we assessed sociodemographic characteristics (gender, age, educational attainment, family status).

Statistical analysis

Statistical analyses were performed with R version 3.6.3 and its package "stats" if not stated otherwise. We examined whether there are significant differences between vignettes with regard to the willingness to address different types of trauma in a conversation using the nonparametric H-test by Kruskall-Wallis as a global test. In case of statistical significance this was followed by a pairwise comparison of the vignettes using Conover's test of mean rank sums with Holm's correction for multiple testing from the R-package "PMCMR". With the same approach, we examined whether the level of stereotyping differs between vignettes, and whether the desire for social distance differs according to the type of trauma mentioned.

To test for possible relations between stereotypes, social distance and avoidance of trauma in conversations, we used regression analyses, including personal trauma experience and contact to persons with trauma experience as additional independent variables. In particular, we used multiple linear regressions to test whether positive or negative stereotypes, previous contact, or personal experience of CT are associated with desire for social distance from a person with CT experience. Using logistic regression analysis, we also tested whether willingness to talk about someone's trauma experience is related to social distance, stereotypes, contact, and own trauma experience. For the logistic model, willingness to talk was collapsed further into two categories representing 1 "willing to talk about the neighbor's trauma experience" and 0 "undecided or unwilling to talk about the neighbor's trauma experience".

In the regression analyses, we focused on the vignettes on childhood sexual abuse and childhood physical abuse, since they are at the core of our research question (H1, H2). To further reduce complexity in these analyses, we conducted a principal component analysis

with all inverted 5-point stereotype items with acceptable Kaiser-Mayer-Olkin (KMO) values (>0.7) using the R-package "psych". The item on being permanently harmed was excluded (KMO=0.55). Two factors with Eigenvalues >1 emerged, depicting negative (Eigenvalue, 2.47) and positive (Eigenvalue, 1.46) stereotypes and cumulatively explaining 44% of the variance. After varimax rotation without Kaiser-normalization from the R-package "GPArotation", we calculated factor scores for both factors with higher values representing stronger agreement with positive or negative stereotypes, respectively. In sensitivity analyses, we conducted a mediation analysis using structural equation modeling, to examine social distance as a mediator linking negative stereotypes to a lower willingness to reach out to trauma victims. The mediation model incorporated negative and positive stereotypes as latent exogenous variables (predictors), social distancing as latent endogenous variable (mediator), and willingness to talk to a victim as manifest endogenous variable (criterion).

The significance level was set at $p < 0.05$.

Results

Regarding contact and personal experience of CT, 34.2% of respondents reported knowing someone having experienced childhood sexual abuse, 43.0% someone with child physical abuse, 38.8% someone with a childhood accident, and 11.4% someone being physically abused as an adult. 10.1% of respondents reported own childhood physical abuse (men, 8.9%; women, 11.1%), and 10.7% reported childhood sexual abuse (men, 5.6%, women, 15.5%).

Asked whether they would talk to their neighbor about their traumatic experience, 45.4% indicated this was unlikely in the case of child sexual abuse, 38.2% in the case of child physical abuse, 30.6% in the case of a childhood car accident, and 24.5% in the case of adult

physical abuse (Figure 1). Kruskal-Wallis rank sum test found significant differences between vignettes ($H(3)=40.5$, $p<0.001$). Conover's post hoc test (with Holm's correction) found partial confirmation of our hypotheses: While the taboo surrounding childhood sexual abuse (mean rank=591.8) and childhood physical abuse (mean rank=605.0) did not differ significantly (H1: $p=.63$), both childhood sexual and physical abuse were surrounded by greater taboo than childhood accidental trauma (mean rank=684.3; H2: $p=0.003$ and $p=0.011$), and both childhood sexual and physical trauma were a more frequently avoided topic than adult physical trauma (mean rank=745.7, H3: $p<0.001$ for each comparison). Fig 1 suggests that the main difference between childhood sexual and physical abuse is that sexual abuse elicits fewer neutral responses, corresponding to slightly more people being both ready and reluctant to talk about childhood sexual compared to physical trauma.

Fig 1. Readiness to address trauma when talking to a person with traumatic experiences. Percentage of respondents willing, undecided or unwilling to address trauma when talking to a neighbor with traumatic experiences. Scenario 1: Childhood sexual abuse. Scenario 2: Childhood physical abuse. Scenario 3: Childhood accident. Scenario 4: Adult physical abuse. Representative population survey, n=1320.

Table 2 shows the agreement with positive and negative stereotypes across vignettes. While most respondents agreed with positive, and disagreed with negative stereotypes, some patterns emerge: The most frequently endorsed negative assumption was that people are permanently harmed by their traumatic experiences. Of the respondents, 61.5% agreed with this statement for child sexual abuse, while only 14.8% disagreed. Agreement followed a clear gradient consistent with our hypotheses H1-H3: Childhood sexual abuse was seen most damaging (all pairwise comparisons $p< 0.005$), followed by childhood physical abuse (50.9%), adult physical abuse (42.9%) and childhood accident (30.9%).

Table 2. Prevalence of negative and positive stereotypes about people with different traumatic experiences.

People who have experienced…								
		Childhood sexual abuse	Childhood physical abuse	Childhood accident	Adult physical abuse	H (df=3)	p	Post hoc
…are harmed for the rest of their lives.	agree	61.5	50.9	30.9	42.9	61.8	<.001	1>2, 1>3, 1>4, 2>3, 3<4
	undecided	23.7	26.2	38.0	34.0			
	disagree	14.8	22.8	31.2	31.1			
	Mean rank	755.2	673.9	544.3	632.5			
…have a higher risk of becoming a criminal.	agree	18.2	21.5	17.7	16.0	15.8	.001	2>3, 2>4
	undecided	21.9	26.8	15.0	19.4			
	disagree	59.9	51.7	67.3	64.6			
	Mean rank	655.9	707.8	615.4	625.1			
…are to some extent guilty of what has happened to them.	agree	7.1	11.1	9.7	8.1	19.6	<.001	1<2, 1<3, 1<4
	undecided	3.4	7.1	11.2	16.5			
	disagree	89.5	81.7	79.1	75.5			
	Mean rank	596.4	647.1	660.7	680.3			
…are unable to have a stable relationship.	agree	24.1	20.6	19.9	22.2	10.1	.018	1>3
	undecided	30.2	34.1	21.5	27.5			
	disagree	45.7	45.3	58.6	50.3			
	Mean rank	674.2	664.9	595.8	644.9			
…are unpredictable.	agree	17.7	19.7	15.4	14.3	7.9	.049	/
	undecided	18.9	27.2	22.8	24.0			
	disagree	63.4	53.1	61.7	61.7			
	Mean rank	628.3	688.2	631.2	628.6			
… must have already been vulnerable before the event, should they develop a mental illness.	agree	17.6	22.5	18.5	18.4	5.1	.16	/
	undecided	21.0	23.8	21.9	26.9			
	disagree	61.4	53.8	59.6	54.7			
	Mean rank	613.8	667.0	626.1	651.0			
…are able to have good friendships.	agree	69.8	61.4	78.6	68.9	23.8	<.001	2<3, 3>4
	undecided	23.5	28.6	17.1	22.5			
	disagree	6.8	10.0	4.3	8.6			
	Mean rank	654.9	598.0	713.2	646.2			
…are just as suitable for a responsible job like any other person.	agree	83.3	76.6	72.8	71.7	13.8	.003	1>3, 1>4
	undecided	11.2	16.6	20.2	20.7			
	disagree	5.5	6.8	7.0	7.6			
	Mean rank	701.3	658.5	634.7	627.4			
…perform their parental duties just as well as other people.	agree	76.1	70.8	74.7	66.3	9.3	.026	1>4
	undecided	17.1	23.6	19.4	24.6			
	disagree	6.8	5.6	5.9	9.1			
	Mean rank	674.1	644.5	667.5	610.6			
…have survived a crisis and have grown through it.	agree	64.7	69.3	77.0	68.1	11.5	.009	1<3
	undecided	29.4	24.5	18.1	26.1			
	disagree	5.9	6.1	4.9	5.8			
	Mean rank	619.4	647.3	696.7	640.3			

Caption: Results of the Kruskal-Wallis rank sum test (H-statistic and p-value) and significant pairwise comparisons (Conover's post hoc test with Holm's correction for multiple testing).

Other stereotypes also showed differences between scenarios, but with varying patterns. People who experienced sexual abuse in childhood are regarded more frequently to be unable to have a stable relationship (endorsed by 24.1% of respondents), the difference being significant (p=0.021), compared to the scenario mentioning a childhood accident (endorsed by 19.9%), and less frequently are they expected to have grown from their

adverse childhood experiences (64.7% vs. 77.0%, childhood accident, $p=0.007$). However, in some respects, they are also seen more favorable than people with other types of trauma: With regard to blame, people reporting childhood sexual abuse are regarded somewhat less guilty than people reporting all other types of trauma (7.1% vs. 8.1-11.1%, all $p<0.05$), and they are seen more competent as parents ($p=0.035$) and in difficult jobs ($p=0.005$), compared to someone reporting adult physical abuse.

People who suffered childhood physical abuse are seen at greater risk of committing criminal offenses (endorsed by 21.5%), a significant difference to childhood accident and adult physical trauma (16.0-17.7%, $p<0.01$ for both comparisons). They are also seen less capable of having good friendships (61.4% vs. 78.6% childhood accident, $p<0.001$). People who have had a severe accident in childhood are generally seen the least negatively, for example, only 30.9% (compared to 42.9-61.5%, all $p<0.01$) see them as permanently harmed, and 78.6% (compared to 61.4-69.8%) trust them to be able to have good friendships.

The desire for social distance did not show large differences between the four scenarios, and followed an opposite pattern: it was lowest towards a person having experienced childhood sexual abuse (median 1.83, interquartile range IQR=1.17) and highest towards a person having suffered from adult physical abuse (median 2.17, IQR 1.17), with childhood accidental trauma and childhood physical abuse (median 2.00, IQR 1.17 each) positioned in the middle. Kruskal-Wallis rank sum test confirmed an overall difference between vignettes ($H=17.1$, $p<0.001$). Conover's test for multiple comparisons of mean rank sums (with Holm's correction) confirmed that social distance was significantly lower in childhood sexual abuse compared to childhood physical abuse ($p=0.019$), childhood accidental trauma ($p=0.019$) and adult physical trauma ($p<0.001$).

Next, we tested whether negative stereotypes regarding childhood sexual and physical trauma are associated with stronger social distance, and whether social distance is associated with less willingness to talk about traumatic experiences (H4).

Table 3 shows bivariate correlations between readiness to raise the topic of trauma in a conversation, desire for social distance, positive and negative stereotypes, contact and personal experience, for the childhood sexual abuse and childhood physical abuse scenario.

Table 3. Pairwise correlation coefficients of willingness to talk, stigma variables, personal experience and contact for childhood sexual abuse (white) and childhood physical abuse (grey).

	Willingness to talk	Social distance	Negative stereotypes	Positive stereotypes	Contact	Reported CSA	Reported CPA
Willingness to talk		-0.15	0.12	0.11	0.14	0.13	-0.06
Social distance	**-0.22****		**0.25*****	**-0.42*****	**-0.19***	-0.02	0.05
Negative stereotypes	0.06	**0.29*****		0.06	-0.06	0.04	0.09
Positive stereotypes	-0.01	**-0.34*****	0.04		**0.19***	0.02	-0.03
Contact	**0.18***	**-0.21*****	-0.07	0.07		0.14	0.14
Reported childhood sexual abuse	**0.17***	0.00	0.14	0.11	**0.26*****		0.08
Reported childhood physical abuse	0.04	-0.02	-0.05	0.11	0.13	**0.21****	

Pearson's *r*, pointbiserial correlation, or Pearson's Φ respectively, with Holm correction for multiple testing. *p<0.05; **p<0.01; ***p<0.001. CSA=childhood sexual abuse; CPA=childhood physical abuse.

For the child sexual trauma scenario, willingness to talk about CT was related to lower social distance, knowing someone affected (contact), and own reported childhood sexual abuse. Contact was related to lower social distance, and more positive stereotypes about a person with childhood physical abuse experience.

Table 4 shows results of a linear regression analysis relating the desire for social distance to negative and positive stereotypes, knowing a person with a similar story, and personal experience, for childhood sexual abuse and childhood physical abuse. The analysis confirmed the bivariate correlations: Negative stereotypes were associated with more, and both

positive stereotypes and contact with less social distance. The models explained 22.3% and 28.8% of the variance of social distance.

Table 4. Predictors of social distance towards a person with childhood trauma (CT). Linear regression analysis, n=284 (Childhood sexual abuse) and n=285 (Childhood physical abuse).

	Childhood sexual abuse		Childhood physical abuse	
	b-value	p	b-value	p
Negative stereotypes	**0.22**	**< 0.001**	**0.24**	**< 0.001**
Positive Stereotypes	**-0.30**	**< 0.001**	**-0.36**	**< 0.001**
Contact (1=yes)	**-0.30**	**0.002**	**-0.19**	**0.046**
Reporting CSA (1=yes)	0.05	0.695	-0.05	0.748
Reporting CPA (1=yes)	-0.05	0.748	0.02	0.913
Gender (1=women)	0.09	0.323	0.00	0.998
Age (years)	0.00	0.056	**0.01**	**0.005**
R^2adj	0.22		0.29	

Table 5 shows logistic regression analyses, predicting odd's ratios of talking to the described neighbor about their experience. Compared to Table 4, social distance is added to the list of predictors. In both childhood sexual abuse and childhood physical abuse, higher social distance was associated with lower odds of talking to the neighbor. Personal experience of childhood sexual abuse increased the likelihood to talk to the person with childhood sexual abuse, while knowing someone with similar experiences increased likelihood to talk to the person with childhood physical abuse. Unexpected independent effects, however, were visible with negative stereotypes: in both scenarios, endorsing more negative stereotypes was associated with greater likelihood of talking to the person described in the vignette, independent from the effect of social distance. Overall, these models explained 11.1% and 11.6% of the variance of willingness to raise the topic of traumatic experiences with the neighbor.

Table 5. Predictors of willingness to talk about childhood interpersonal trauma. Logistic regression analysis, n=281 (Childhood sexual abuse) and n=284 (Childhood physical abuse).

	Childhood sexual abuse (n=281)				Childhood physical abuse (n=284)			
	95% CI for odds ratio			p	95% CI for odds ratio			p
	Lower	Odds ratio	Upper		Lower	Odds ratio	Upper	
Social distance	**0.35**	**0.52**	**0.77**	**0.002**	**0.44**	**0.66**	**0.96**	**0.033**
Negative stereotypes	**1.06**	**1.43**	**1.94**	**0.021**	**1.22**	**1.64**	**2.22**	**0.001**
Positive stereotypes	0.72	0.98	1.33	0.877	0.93	1.26	1.72	0.135
Contact (1=yes)	0.80	1.40	2.46	0.240	**1.06**	**1.85**	**3.24**	**0.031**
Reported CSA (1=yes)	**1.18**	**2.60**	**5.84**	**0.018**	0.63	1.59	3.95	0.321
Reported CPA (1=yes)	0.25	0.67	1.67	0.404	0.21	0.56	1.39	0.226
Gender (1=women)	0.58	0.99	1.69	0.981	0.92	1.58	2.75	0.098
Age (years)	**0.96**	**0.98**	**0.99**	**0.008**	0.99	1.01	1.02	0.512

Note. Childhood sexual abuse: Model $\chi^2(8) = 32.6$, p = 0.00007, $R^2 = 0.11$ (Cox-Snell), 0.15 (Nagelkerke). Childhood physical abuse: Model $\chi^2(8) = 34.8$, p = 0.00003, $R^2 = 0.12$ (Cox-Snell), 0.16 (Nagelkerke).

Due to the unexpected independent effects of negative stereotypes in the childhood physical and sexual abuse logistic regression models, we conducted further sensitivity analyses of H4 using structural equation modeling. The specification of the mediation model, incorporating negative and positive stereotypes as latent exogenous variables, social distancing as latent endogenous variable, and willingness to talk to a victim as manifest endogenous variable, is presented in Fig 2 (please also see Supporting information, S1-S2 Figs and S1-S3 Tables). In the full sample, negative stereotypes about trauma victims increased the general desire for social distance from affected persons (B=.17, p<.001), which, in turn, decreased the inclination to talk to an afflicted individual (B=-.31, p< .001). The resulting mediation effect, linking negative stereotypes to a reduced willingness to talk to trauma victims (H 4), was statistically significant (B=-.05, p < .001; S3 Table). However, there was also an unexpected direct effect in participants agreeing with negative stereotypes, that increased their willingness to talk to trauma victims (B=.14, p=.002). In total, the adverse mediated effect and beneficial direct effect effectively canceled each other out (B=.09, p =.056; S3 Table).

Fig 2. Structural equation model specification for the proposed relationship between stereotypes, social distancing, and tabooing behaviour. The measurement part (left) shows the operationalization of the two exogenous latent predictors (ξ_1, ξ_2, operationalized by exogenous indicators x_1-x_9; item labels q_1-q_9 according to Table 1) and the endogenous latent mediator (η_1, operationalized by exogenous indicators y_1-y_6). Arrows represent parameters (measurement errors δ_1-δ_9 or ε_1-ε_6, factor loadings λ_1^x-λ_9^x or λ_1^y-λ_6^y, and factor covariance φ_{12})–dashed if they were fixed. The structural part (right) shows the endogenous criterion (y_7), its measurement error (ε_7), the standardized path coefficients relating it to the exogenous (γ_1- γ_4) and endogenous (β_1) latent variables (labels a-d according to Table 2), and the residuum of the mediator (ζ_1).

Discussion

In this first study examining the public stigma of CT, we found considerable reluctance to reach out to someone indicating they were still struggling with traumatic childhood experiences. People were particularly unwilling to talk about childhood sexual and physical trauma, as opposed to childhood accidental trauma and adult physical trauma. Although the person in the vignette themselves mentioned their traumatic experience, and hinted at ongoing related difficulties, only one in three respondents considered actively raising this topic with them again, compared to one in two in adult physical trauma. Our results thus confirm the difficulties surrounding conversations about CT experiences among the general public.

We hypothesized that a possible reason for a reluctance to address trauma was stigma, which was confirmed by our findings. Desire for social distance, an established measure of individual discrimination (22), was related to lower willingness to talk about CT, while negative stereotypes were associated with more, and positive stereotypes with less social distance. Negative and positive stereotypes showed a plausible pattern, with childhood physical trauma being associated with lower expectations regarding friendships, and stronger suspicion of criminal offenses, and childhood sexual trauma evoking particularly strong notions of lasting harm and difficulties in partnerships. Hence, there are indications

that a stigma of CT exists, and that it does in fact contribute to the taboo surrounding CT. Different to our assumptions, however, the desire for social distance did not show the expected gradient from interpersonal to accidental, and from childhood to adult trauma. Instead, childhood sexual abuse provoked the least desire for social distance. In some respect, childhood sexual abuse also provoked more positive responses compared to the other scenarios, for example with regard to being blamed for the trauma. Hence, stigma does not seem to be the only dynamic guiding reactions to persons with CT.

Another indication on the complexity of reactions towards persons with CT is our finding that negative stereotypes were related to greater willingness to talk about CT. This was only visible in multiple regression analyses when controlling, among other variables, for social distance, and the finding was replicated in mediation analysis. The most likely explanation for these findings is that beyond stigma, other motives guide willingness to talk about traumatic events. Possibly, endorsing negative stereotypes is also related to notions like taking the problem seriously and not trivializing the consequences of childhood abuse. Then, endorsing negative stereotypes could be related to worrying about the person or pitying them, which could correspond to greater openness to talk about what had happened to them.

To our knowledge, this is the first study examining public stigma with regard to CT. Considering its strengths and limitations, the lack of a measure of positive and negative emotions is probably its most severe shortcoming. Theoretically, positive emotions like empathy could also be related to greater reluctance to address trauma, reflecting a desire to protect the person or to avoid hurting them by raising a painful topic. Examining the interplay of emotions, stigma and taboo when dealing with reports of CT survivors, is thus a desideratum for future research. Among the strengths of our study are its representative

general population sample and the use of the short scenarios of an identical situation pertaining to four different types trauma, which enabled us to compare public reactions to these different types.

Our study fills an obvious gap in trauma related stigma research. So far, the stigma associated with trauma has mainly been explored with regard to stigma experience rather than public expressions of stigma. Moreover, previous research on the stigma of childhood abuse has put a strong focus on survivors of childhood sexual trauma. We found survivors of childhood physical abuse stigmatized to a similar degree, evoking even higher desire for social distance compared to survivors of childhood sexual abuse. This group of victims has so far received considerably less attention in stigma research.

Given the role of social support in preventing adverse long-term outcomes of childhood abuse (23, 24), and the role of shame, stigma and devaluation in further victimizing the survivors of childhood abuse, focusing on the stigma of being traumatized itself seems justified. It should be noted, however, that in many survivors of childhood adversities, multiple stigmatizing conditions might overlap, like having developed a mental illness or substance use disorder (25), leading to intersectional stigma (26).

Improving the way we interact with victims of CT could ameliorate the adverse outcomes of trauma. The fact that survivors of childhood sexual trauma were more likely to talk to a childhood sexual trauma victim in our study suggests that from a victim's perspective, engaging is considered more helpful than staying away. However, our study shows that stigma might not be sufficient to explain hesitation to engage with trauma victims. The interplay of negative stereotypes, social distance, fear of causing new harm, emotions and desire to help seems thus a valuable subject for future studies. Understanding why people are reluctant to talk to someone who signals they are in need for help for past traumatic

experiences will pave the way for interventions that increase social support for people with a history of CT.

References

1. Danese A, Baldwin JR. Hidden Wounds? Inflammatory Links Between Childhood Trauma and Psychopathology. Annu Rev Psychol. 2017;68:517-44.
2. Curran E, Adamson G, Rosato M, De Cock P, Leavey G. Profiles of childhood trauma and psychopathology: US National Epidemiologic Survey. Soc Psychiatry Psychiatr Epidemiol. 2018;53(11):1207-19.
3. Hovens JG, Wiersma JE, Giltay EJ, van Oppen P, Spinhoven P, Penninx BW, et al. Childhood life events and childhood trauma in adult patients with depressive, anxiety and comorbid disorders vs. controls. Acta Psychiatr Scand. 2010;122(1):66-74.
4. Chapman DP, Whitfield CL, Felitti VJ, Dube SR, Edwards VJ, Anda RF. Adverse childhood experiences and the risk of depressive disorders in adulthood. J Affect Disord. 2004;82(2):217-25.
5. Lieb K, Zanarini MC, Schmahl C, Linehan MM, Bohus M. Borderline personality disorder. Lancet. 2004;364(9432):453-61.
6. Matheson SL, Shepherd AM, Pinchbeck RM, Laurens KR, Carr VJ. Childhood adversity in schizophrenia: a systematic meta-analysis. Psychol Med. 2013;43(2):225-38.
7. Kennedy AC, Prock KA. "I Still Feel Like I Am Not Normal": A Review of the Role of Stigma and Stigmatization Among Female Survivors of Child Sexual Abuse, Sexual Assault, and Intimate Partner Violence. Trauma Violence Abuse. 2018;19(5):512-27.
8. Overstreet NM, Willie TC, Sullivan TP. Stigmatizing Reactions Versus General Negative Reactions to Partner Violence Disclosure as Predictors of Avoidance Coping and Depression. J Interpers Violence. 2019;34(8):1734-52.
9. Murray CE, Crowe A, Overstreet NM. Sources and Components of Stigma Experienced by Survivors of Intimate Partner Violence. J Interpers Violence. 2018;33(3):515-36.
10. Feiring C, Simon VA, Cleland CM. Childhood sexual abuse, stigmatization, internalizing symptoms, and the development of sexual difficulties and dating aggression. J Consult Clin Psychol. 2009;77(1):127-37.
11. Goffman E. Stigma; notes on the management of spoiled identity. Englewood Cliffs, N.J.,: Prentice-Hall; 1963. 147 p. p.
12. Link BG, Phelan JC. Conceptualizing stigma. Annual Review of Sociology. 2001;27:363-85.
13. Evans-Lacko S, Brohan E, Mojtabai R, Thornicroft G. Association between public views of mental illness and self-stigma among individuals with mental illness in 14 European countries. Psychol Med. 2012;42(8):1741-52.
14. Corrigan PW, Rafacz J, Rusch N. Examining a progressive model of self-stigma and its impact on people with serious mental illness. Psychiatry Res. 2011;189(3):339-43.
15. Finkelhor D, Browne A. The traumatic impact of child sexual abuse: a conceptualization. Am J Orthopsychiatry. 1985;55(4):530-41.
16. Ackerman PT, Newton JE, McPherson WB, Jones JG, Dykman RA. Prevalence of post traumatic stress disorder and other psychiatric diagnoses in three groups of abused children (sexual, physical, and both). Child Abuse Negl. 1998;22(8):759-74.
17. Knudsen EI, Heckman JJ, Cameron JL, Shonkoff JP. Economic, neurobiological, and behavioral perspectives on building America's future workforce. Proc Natl Acad Sci U S A. 2006;103(27):10155-62.
18. Link BG, Cullen FT, Frank J, Wozniak JF. The social rejection of former mental patients - understanding why labels matter. American Journal of Sociology. 1987;92(6):1461-500.

19. Angermeyer MC, Matschinger H. Violent attacks on public figures by persons suffering from psychiatric disorders. Their effect on the social distance towards the mentally ill. Eur Arch Psychiatry Clin Neurosci. 1995;245(3):159-64.
20. Grabe HJ, Schulz A, Schmidt CO, Appel K, Driessen M, Wingenfeld K, et al. [A brief instrument for the assessment of childhood abuse and neglect: the childhood trauma screener (CTS)]. Psychiatr Prax. 2012;39(3):109-15.
21. Bernstein DP, Stein JA, Newcomb MD, Walker E, Pogge D, Ahluvalia T, et al. Development and validation of a brief screening version of the Childhood Trauma Questionnaire. Child Abuse and Neglect. 2003;27(2):169-90.
22. Link BG, Yang LH, Phelan JC, Collins PY. Measuring mental illness stigma. Schizophrenia Bulletin. 2004;30(3):511-41.
23. Holz NE, Tost H, Meyer-Lindenberg A. Resilience and the brain: a key role for regulatory circuits linked to social stress and support. Mol Psychiatry. 2020;25(2):379-96.
24. Runtz MG, Schallow JR. Social support and coping strategies as mediators of adult adjustment following childhood maltreatment. Child Abuse Negl. 1997;21(2):211-26.
25. Stolzenburg S, Tessmer C, Corrigan PW, Böttge M, Freitag S, Schäfer I, et al. Childhood trauma and self-stigma of alcohol dependence: Applying the progressive model of self-stigma. Stigma and Health. 2018;3(4):417.
26. Oexle N, Corrigan PW. Understanding Mental Illness Stigma Toward Persons With Multiple Stigmatized Conditions: Implications of Intersectionality Theory. Psychiatr Serv. 2018;69(5):587-9.

Supporting information

S1 Table. Rotated factor loadings. Results of the principal components analysis with varimax rotation and without Kaiser-normalization, h^2 communality.

S2 Table. Global model fit indices of the mediation model in the full sample (single-group structural equation modelling - SGSEM) and per vignette (multi-group - MGSEM) using a weighted least squares mean- and variance-adjusted estimator and pairwise or listwise deletion of missing values, respectively. CFI comparative fit index, CI confidence interval, *df* degrees of freedom, *N* sample size, RMSEA root mean square error of approximation, SRMR standardized root mean square residual.

S3 Table. Results of mediation analysis. Standardized path coefficients (Beta values) and significance level (p) of the mediation analysis in the full sample (single-group structural equation modelling - SGSEM) and per vignette (multi-group - MGSEM) using a weighted least squares estimator and list-wise or pairwise deletion of missing values, respectively. For the path coefficient labels a – d please refer to Figure 2. * < .05, ** < .01, *** < .001.

S1 Fig. Path coefficients of the MGSEM analysis for vignette “Childhood sexual trauma”. Weighted least squares estimated path coefficients (standardized). The parameter estimates are represented by the opacity level of the arrows.

S2 Fig. Path coefficients of the MGSEM analysis for vignette “Childhood physical trauma”. Weighted least squares estimated path coefficients (standardized). The parameter estimates are represented by the opacity level of the arrows.

Figure 1

Click here to access/download;Figure;Figure 1.tif

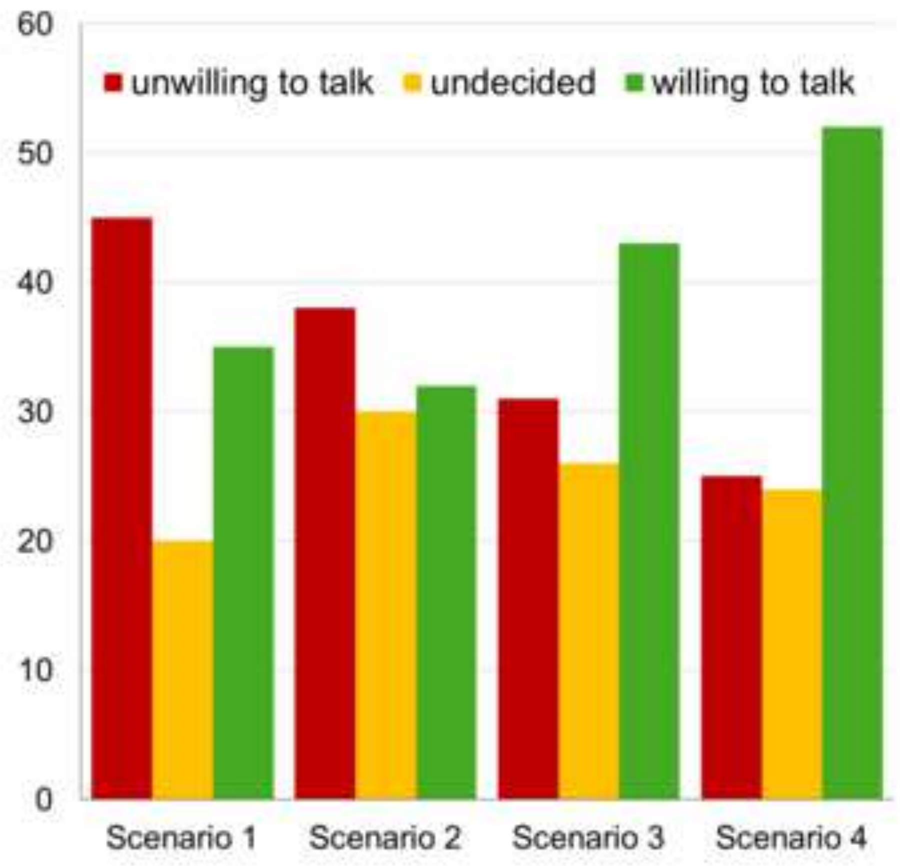

Figure 2

Click here to access/download;Figure;Fig 2.tif

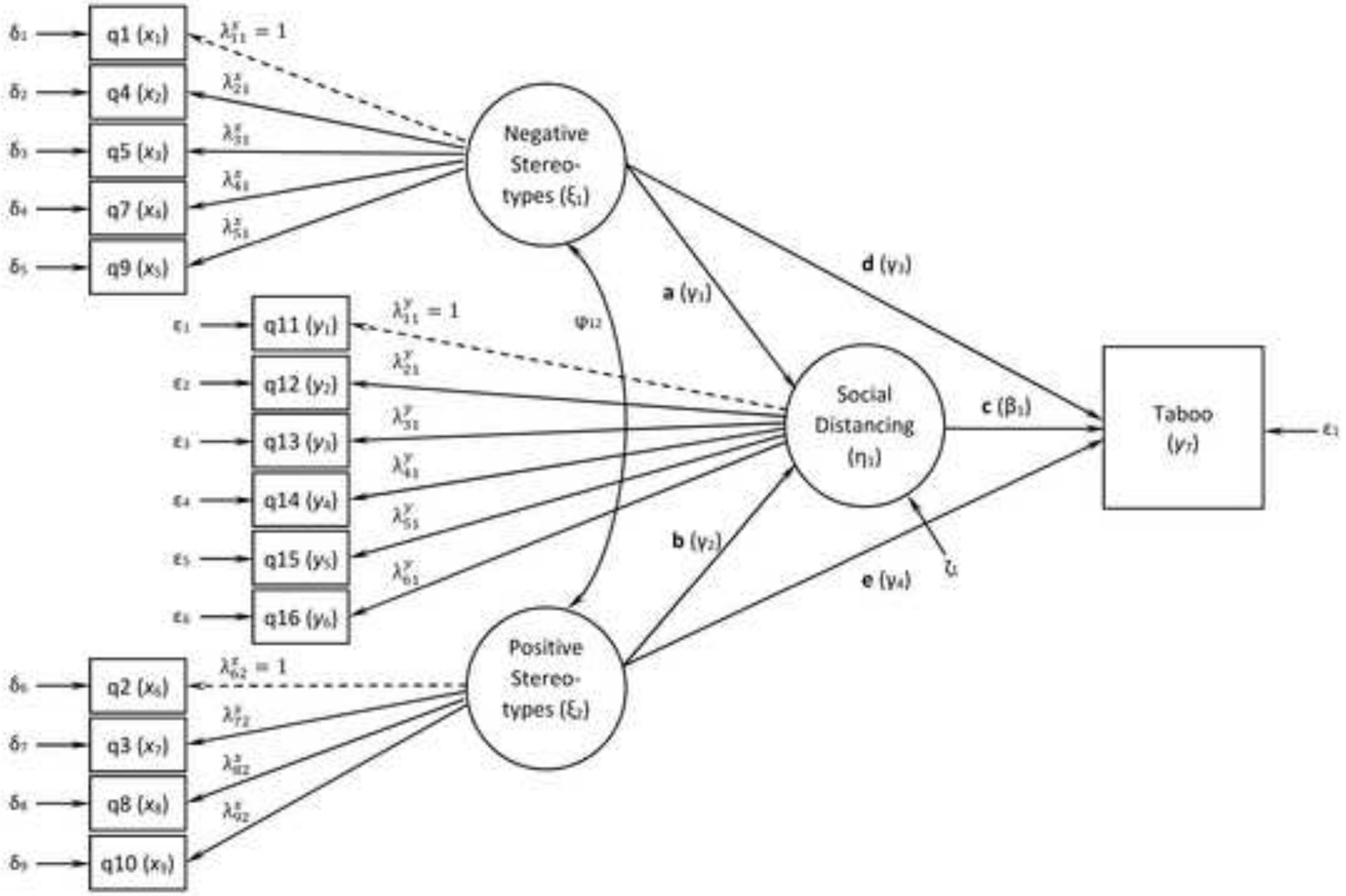

Table S1

Click here to access/download
Supporting Information
S1 Table.docx

Table S2

Click here to access/download
Supporting Information
S2 Table.docx

Table S3

Click here to access/download
Supporting Information
S3 Table.docx

Figure S1

Click here to access/download
Supporting Information
Fig S1.tif

Figure S2

Click here to access/download
Supporting Information
Fig S2.tif
