# Supplementary material for: Stigma as a barrier to addressing childhood trauma in conversation with trauma survivors: A study in the general population
Source: PLoS One. 2021 Oct 18;16(10):e0258782. doi: 10.1371/journal.pone.0258782 (PMC8523057; doi:10.1371/journal.pone.0258782)
Supplement: Attachment — Submitted filename: Response to reviewers2.docx [file pone.0258782.s008.docx]

We thank the 2nd reviewer for his careful reading of our revision and for his thought provoking critique of our paper. We acknowledge the points the reviewer raised, but despite his/her pessimisitc view of the paper, we feel confident to address these concerns.

*I appreciate the authors' responsiveness to reviewer feedback. In particular, the hypotheses are more clearly described in the Introduction; there is more evidence of citing and engaging with existing research on childhood trauma stigma; and the rationale for the Neighbor scenario is clearer, if still confounded, to my mind, with the neighbor/trauma survivor violating social norms by disclosing on a first meeting.  Despite the authors' commendable, detailed work in revising the manuscript and explaining their methods and statistical results, there remain experimental confounds in the design of the study that cannot be addressed post-hoc.  Specifically:*

(1) *The authors' data cannot address Hypothesis 2, because trauma type (accidental vs. interpersonal) is confounded with age at which trauma occurred (the only accidental trauma vignette is in childhood), and*
(2) *the data cannot address Hypothesis 3 as it is currently stated, because adulthood trauma is confounded with physical abuse trauma, such that the authors are not comparing childhood vs. adult abuse per se but rather childhood sexual, physical, and accidental trauma vs. adulthood physical trauma.*

Response: The focus of our study is on childhood trauma. So we introduced childhood accidental trauma to contrast it with both childhood physical and sexual trauma. We have made this more clear in our introduction:

“Further, we hypothesize that childhood interpersonal trauma is associated with more stigma than childhood accidental trauma (H2).”

Similarly, we contrast childhood physical trauma to adult physical trauma, to see whether the age of the person in the vignette makes a difference. “Since adults are perceived as more stable in order to overcome traumatic events, while children are regarded as more vulnerable and more profoundly affected by trauma, we assume that childhood physical trauma triggers more stigma than adult physical trauma (H3).”

While we avoid the confounding suspected by the reviewer by making our focus more specific, we acknowledge that any possible difference between perceptions of adult and child trauma survivors could be dependent on type of trauma, and have acknowledged this in our limitations section:

“Any difference between reactions to an adult or childhood trauma victim are elicited with regard to physical, but not sexual abuse.” (p. 25)

(3) *I appreciate the constraints on statistical power and experimental conditions that can be imposed by limited financial funding for a project, but given that, the experimental design needs to be adjusted accordingly. Additionally, it is hard to follow both the rationale for and the statistical process for testing Hypothesis 4, in that here the authors drop the issue of childhood vs. adulthood and accidental vs. impersonal traumas and examine stigma and trauma broadly as constructs in the SEM model,*

Response: We have made now clear when stating H4 why we drop the childhood/adult distinction here for a general analysis of the stigma process with regard to trauma survivors (and use the four vignettes again in an explorative multi-group SEM afterwards, see response to (5)).

“Finally, to see to what extent the concept of stigma applies to public reactions to trauma survivors, we assumed that in accordance with established models of stigma (15), negative stereotypes about people with trauma experience increase the general desire for social distance from such persons, which ultimately manifests as a greater reluctance to talk to them about their trauma experience (H4). “

(4) *and they also introduce the concept of positive stereotypes which had not been mentioned previously in the introduction (until the statement of H4), making it hard to follow the thread from theory to method to data analysis.*

Response: We introduce the negative and positive stereotypes elicited in our methods section on p. 7.

(5) *Moreover, after adding a more detailed explanation of the statistical analyses in the Results section, as requested during the review process, it is now clearer how exquisitely complex the analyses are for H4, presenting barriers to the dissemination of the authors' work, when it is so difficult to parse the multi step process of this analysis.*

Respones: We agree that our description of our SEM is complex, and somewhat hides the pretty straight forward theoretical approach that was tested with these models. We have have outlined in our H4 that we tested the established stigma process with stereotypes, social distance and resulting discrminatory behavior (see response (3)). We now add at the beginning of the Results section reporting the SEM:

“We used a single-group SEM (SGSEM) to test the interrelation of the stigma components in the entire sample, and exploratively used multi-group SEM (MGSEM) to test these interrelations for each vignette.”

In fact, the unexpected finding that negative stereotypes were directly associated with more willingness to talk to the trauma victim justifies the explorative approach looking at each vignette, showing that this was particularly the case in childhood interpersonal trauma. By reporting these unexpected findings, our study shows that stigma is a relevant, but not exhaustive concept explaining the taboo surrounding CT. We discuss this on page 25.

(6) *I realize this assessment might be disappointing to the authors who have clearly put a lot of preparation into their manuscript. But in a society already predisposed to stigma and doubt surrounding childhood trauma, I think it's all the more important that trauma research methods be rigorously designed and consistent with principles of open science such as pre-registration of hypotheses and analysis plans. Thank you for the opportunity to review this manuscript.*

Response: We respectfully disagree. Our findings do not diminish or question the harm done by stigma and doubt surrounding childhood trauma, but for the first time use a representative population sample to examine and understand it. Our methodology and statistical analysis are sound, and our research gives some answers, while also posing new questions.
